# Microdebrider is less aerosol-generating than CO_2_ laser and cold instruments in microlaryngoscopy

**DOI:** 10.1007/s00405-021-07105-9

**Published:** 2021-10-08

**Authors:** Enni Sanmark, Lotta-Maria A. H. Oksanen, Noora Rantanen, Mari Lahelma, Veli-Jukka Anttila, Nina Atanasova, Antti-Pekka Hyvärinen, Teemu Kinnari, Ahmed Geneid

**Affiliations:** 1 Faculty of Medicine, University of Helsinki, Valhallankatu 7a 21, 00250 Helsinki, Finland; 2 Department of Otorhinolaryngology and Phoniatrics-Head and Neck Surgery, Helsinki University Hospital, Helsinki, Finland; 3 Faculty of Science, Mathematics and Statistics, University of Helsinki, Helsinki, Finland; 4 HUS Inflammation Center, Helsinki University Hospital, Helsinki, Finland; 5 Faculty of Biological and Environmental Sciences, Molecular and Integrative Biosciences, University of Helsinki, Helsinki, Finland; 6 Finnish Meteorological Institute, Helsinki, Finland

**Keywords:** COVID-19, Airborne transmission, Laryngology, CO_2_ laser, Microdebrider, Cold instruments, Microlaryngeal surgery

## Abstract

**Objective:**

COVID-19 spreads through aerosols produced in coughing, talking, exhalation, and also in some surgical procedures. Use of CO_2_ laser in laryngeal surgery has been observed to generate aerosols, however, other techniques, such cold dissection and microdebrider, have not been sufficiently investigated. We aimed to assess whether aerosol generation occurs during laryngeal operations and the effect of different instruments on aerosol production.

**Methods:**

We measured particle concentration generated during surgeries with an Optical Particle Sizer. Cough data collected from volunteers and aerosol concentration of an empty operating room served as references. Aerosol concentrations when using different techniques and equipment were compared with references as well as with each other.

**Results:**

Thirteen laryngological surgeries were evaluated. The highest total aerosol concentrations were observed when using CO_2_ laser and these were significantly higher than the concentrations when using microdebrider or cold dissection (*p* < 0.0001, *p* < 0.0001) or in the background or during coughing (*p* < 0.0001, *p* < 0.0001). In contrast, neither microdebrider nor cold dissection produced significant concentrations of aerosol compared with coughing (*p* = 0.146, *p* = 0.753). In comparing all three techniques, microdebrider produced the least aerosol particles.

**Conclusions:**

Microdebrider and cold dissection can be regarded as aerosol-generating relative to background reference concentrations, but they should not be considered as high-risk aerosol-generating procedures, as the concentrations are low and do not exceed those of coughing. A step-down algorithm from CO_2_ laser to cold instruments and microdebrider is recommended to lower the risk of airborne infections among medical staff.

## Introduction

During the COVID-19 pandemic the evidence is mounting that spreading by inhalation of virus-laden aerosols is a significant transmission mode indoors, particularly when aerosol-generating procedures (AGPs) are performed [1–3]. The generation of aerosol particles in surgical procedures has been addressed in ear, nose, and throat (ENT) specialties, as virus load is known to be high in the nasal and upper respiratory tract area, which is often manipulated mechanically and instrumentally during ENT examinations and procedures [4]. In addition, ENT doctors are highly likely to meet potential COVID-19 patients since its spectrum of symptoms is quite similar to other ENT-related infections: cough, common cold, hoarseness of voice, fever, anosmia, and sore throat [5].

Airborne transmission of microorganisms and viruses can occur either via droplets or aerosols. Droplets that are likely to settle proximate to the source have been defined as particles bigger than 5 µm [6, 7]. However, in recent statements, aerosol scientists have suggested moving the boundary up to 100 µm particles, as even particles this size can remain in the air for longer periods of time if the settling velocity is exceeded by the velocity of air moving throughout a room [8]. Aerosols, and especially fine aerosols smaller than 5 µm, remain in the indoor air normally up to several hours. However, as in the case of droplets, environmental factors also have a significant impact. Aerosols concentrate near the source, but because their ability to stay in the air for a long time and spread in the space they create considerable challenges for infection control [6]. It is important to note that when we discuss the spread of pathogens the transmission is not only via aerosols or droplets, but by both routes simultaneously. Whether a particle should be classified as a droplet or an aerosol depends on environmental factors such as temperature, barometric pressure, humidity, and air currents.

An infectious dose of viral diseases is poorly understood, and thus, it has not been possible to set a quantitative limit on significant or high-risk aerosol release. Concentration of aerosols generated during coughing has been set as a risk limit for clinically marked aerosol generation during surgical procedures and other aerosol-generating activities [9–11]. Microlaryngoscopies are considered as AGPs and accordingly have been avoided or reduced to urgent cases whenever possible during the peaks of the COVID-19 pandemic [12–14]. In microlaryngoscopies, suspicions about aerosol generation arise from the nature of the operations. Continuous air flow in the larynx, manipulation of the mucosa, and use of suction, CO_2_ laser, monopolar cautery, and microdebrider are thought to cause particle generation [15]. However, views on the effect of both suction and electrocauteries are controversial in terms of production of aerosols and droplets [16–19]. By contrast, the CO_2_ laser has been assumed unanimously to generate aerosols, and the results of Guderian et al. confirm this presumption [20].

The ongoing global pandemic has caused several hospital outbreaks and shown that infection control is challenging and requires special attention. To establish effective infection control measures and personal protection guidelines as well as valid risk assessment, it will be important to understand the generation of aerosols during different hospital activities also after the current COVID-19 pandemic. Aims of our study are to measure aerosol generation during laryngological operations and to assess the operating room (OR) staff exposure to potentially contagious particles during typical laryngological surgeries under general anesthesia.

## Materials and methods

The study was conducted at Helsinki University Hospital, Department of Ear, Nose, and Throat between August and November 2020. Particle measurements were carried out during laryngological operations in the same ORs used in the hospital’s normal routine.

The overall air change rate varied in different ORs between 31.11 and 31.95 changes/h and in the laminar ventilation area between 557 and 572 changes/h, both of which exceed the American Institute of Architects’ guidelines of 25 exchanges/h [21]. The ORs had Recair 4C and INPO-1.5 ventilation systems. The relative humidity during the operations varied between 24.3 and 69.8%.

Particle measurement carried out with an optical particle sizer (OPS) was continuous during the surgeries. The recording frequency applied was 10 s. The study protocol was designed to reflect the exposure received by the operational staff, and thus, OPS was situated on average 136 cm from the head of the patient (range 110–230 cm). The same research nurse attended all recorded surgeries and made accurate notes of all steps of the surgery. Surgeries were performed by five experienced surgeons who all work in the same department and following same protocols.

### Measured laryngological operations

Overall, 13 laryngeal operations under general anesthesia, including biopsies or lesion removals, were measured. All operations started with intubating the patient. Following intubation, a laryngoscope was inserted. For certain patients, a tape and a soft laryngeal cushion was applied to the neck to gain better visualization of the vocal folds by applying external counterpressure. Our protocol included endoscopic imaging of the operative field using a Hopkins rod attached to a high-definition camera. Laryngeal operations were done using cold dissection technique which means using only cold instruments, CO_2_ laser, or microdebrider, or a combination of the forementioned techniques. For the statistical analysis, we divided operations’ phases based on instruments used into three categories (CO_2_ laser, microdebrider, and cold instruments). An operation including a combination of the forementioned techniques had each part of the operation analyzed separately depending on the instrument included.

In surgeries where the CO_2_ laser was used, patients were intubated with a Rusch Laser-Resistant Tracheal Tube, including air and saline-filled cuffs. CO_2_ laser (Lumenis Ltd., Santa Clara, CA, USA) coupled with microscope (400 mm Zeiss) was used to remove tissues such as papilloma or vocal fold polyps. CO_2_ laser in cases of laryngeal papillomatosis was used for cutting and/or evaporating papilloma from the larynx. In cases of polyps and glottal cancer, it was used to excise the lesion. A CO_2_ laser smoke evacuation was applied in all laryngeal CO_2_ laser operations. Microdebrider (Medtronic IPC, speed 1500 rpm, simultaneous suction, no douche) was used in removing the laryngeal papilloma. It is a power blade with a long shaft that is used for removal of tissues. The microdebrider includes suction for removal of tissues.

### Measured references

We used two references in this study: background aerosol concentration and our earlier results on aerosol concentration generated when coughing. Background data were measured separately from clean, empty ORs with the same OPS device used during surgeries. For the coughing reference, altogether 291 coughs were measured from 37 healthy volunteers at distances 40, 70, and 100 cm from the same OPS device used in this study [22]. The coughing data were collected, measured, and analyzed in the same manner as data collected from surgeries.

### Optical particle sizer

To measure the particle concentration and size distributions of generated particles, we used an Optical Particle Sizer (TSI model 3330 OPS). The measurement principle of the device is based on optical light scattering from the particles, and observed particles are in the size range from 0.3 to 10 µm. TSI 3330 OPS used in our study reports the optical size of particles in 16 size bins every 10 s. These size bins have been factory-calibrated with polystyrene latex (PSL) particles having a refractive index of 1.59. The OPS device uses real-time feedback monitoring of the flow rate to ensure concentration accuracy. The same adjustments were also used for background measurements.

The OPS was factory-calibrated prior to the measurements. In addition, the nominal 1 L/min flow rate was audited regularly with a mass flow meter (TSI model 4143) and varied only by ± 2% during the measurements.

### Power of the study

This study combines aerosol physics and medicine. Thus, existing power calculators are not available to calculate the power of this study but in the aerosol physics, three repeated measurements have been estimated to be the minimum number. A similar design has also been used in previous studies. In those studies, the duration of single measurements has been between 0.5 and 5 min and measurements have been repeated zero to 14 times. The total time measured has varied from 2 to 70 min depending on the study and the procedure [20, 23, 24]. In our study, we measured altogether 157.6 min (cold dissection 40.5 min, microdebrider 13.5 min, CO_2_ laser 105.2 min). The duration of a single measurement was between 0.2 and 58.1 min and measurements were repeated 7–16 times. We used the real-time aerosol measurements which consist of single particle detection averaged to 10 s—thus comprising a statistically and temporally comprehensive data set for each individual case-wise data point (*n* = 946: cold dissection *n* = 234, microdebrider *n* = 81, laser *n* = 631). The overall power of this study can be considered adequate.

### Statistical methods

The size-dependent aerosol concentrations measured with OPS were normalized regarding the respective sizing bin widths within 0.3–10 µm. The volume-weighted particle size distribution and total particle concentrations per cubic centimeter were calculated. The particles were categorized based on diameter as follows: < 1, 1–5, and > 5 µm. Mean with standard deviation (SD) was chosen as statistically representative to describe average aerosol exposure during the investigated procedure. Due to infection risk being related to cumulative aerosol exposure, mean was chosen as a central representative instead of median regardless of highly skewed distribution with a large number of zero particle observations. Pairwise comparisons were calculated using unpaired Student’s *t* test. Differences in aerosol concentrations between the techniques were compared using one-way analysis of variance (ANOVA) with Tukey HSD post hoc test for multiple comparisons. For comparisons with the background reference, one-tailed paired-samples Student’s *t* test was used. Prior to comparisons, the data were log10-normalized. The analyses were performed using Microsoft Excel 2016 (Microsoft Corporation, Redmond, WA, USA), and GraphPad Prism version 9.0.2 for Mac (GraphPad Software, San Diego, CA, USA) or RStudio version 1.3.959 (R Foundation for Statistical Computing, Vienna, Austria). A *p* value < 0.05 was considered to be statistically significant.

### Ethical considerations

All procedures that involved human participants were conducted in accordance with the ethical standards of the institutional or national research committee and the 1964 Declaration of Helsinki and its later amendments or comparable ethical standards. The Ethics Committee of Helsinki University Hospital approved the study protocol (HUS/1701/2020). All responders provided written informed consent prior to their participation.

## Results

A total of 13 patients, 61 periods (18 with cold instruments, 36 with CO_2_ laser, and 7 with microdebrider) and 946 datapoints of laryngeal operations were included in the study. The periods were picked manually from the procedure measures so that there were 0, 3–15 min between periods. However, only one interval was less than 1 min. When picking the procedures, we followed a step-up algorithm from cold instruments to the microdebrider and CO_2_ laser which means that cold instruments picked collected only when they were used before the use of microdebrider and CO_2_ laser and microdebrider periods were picked only when it was used before the use of CO_2_ laser. In addition we ensured that, the particle concentration returned to the baseline level before next analyzed period. Patient characteristics and clinical data are presented in Table 1.

**Table 1:** Patient characteristics and measured laryngeal procedures

	Cold instruments	Microdebrider	CO_2_ laser
Number of patients (*n*)^a^	10	4	8
Females (%)	30	25	38
Age, median (range)	66.5 (24‒75)	52 (47‒56)	54.5 (47‒72)
BMI kg/m^2^, mean ± SD (range)	27.8 ± 6.55 (21.5‒44.9)	29.0 ± 9.14 (22.5‒42.3)	30.0 ± 8.67 (22.5‒44.9)
Number of measured procedure periods	18	7	36
Procedure done/diagnosis (ICD-code)	Endoscopic excision of laryngeal papillomatosis/laryngeal papillomatosis (D14.1&B97.7) *n* = 4 Cordotomy of glottal epidermoid carcinoma/glottal epidermoid carcinoma (C32.0) *n* = 1 Endoscopy and biopsy of larynx/other vocal cord disease (J38.3) *n* = 1 Endoscopic excision of lesion of larynx/chronic laryngitis the larynx and polyp on vocal cords (j37.0, J38.1) *n* = 1 Endoscopy and biopsy of larynx/chronic malignant tumor of the glottis (C32.0&) *n* = 1 Endoscopic excision of lesion of larynx/chronic laryngitis (J37.0) *n* = 1 Endoscopic excision of lesion of larynx/tumor of uncertain or unknown nature (D38.0&) of the larynx *n* = 1	Endoscopic excision of laryngeal papillomatosis/benign tumor of the larynx and laryngeal papillomatosis (D14.1&B97.7) *n* = 4	Endoscopic excision of laryngeal papillomatosis/laryngeal papillomatosis (D14.1&B97.7) *n* = 4 Cordotomy of glottal epidermoid carcinoma/glottal epidermoid carcinoma (C32.0) *n* = 1 Endoscopic excision of lesion of larynx/chronic laryngitis Endoscopic excision of vocal fold polyp (J38.1) *n* = 1 Endoscopic excision of lesion of larynx/chronic laryngitis the larynx and polyp on vocal cords (j37.0, J38.1) *n* = 1

^a^Some operations included more than one instrument type (total *n* = 13)

Most particles in all procedures were detected in size class < 1 μm, while only a very low concentration of particles > 5 μm was seen. All background concentrations were very low (maximum mean total concentration 0.008 particles/cm^3^), enabling the accurate evaluation of particle concentration generated during the procedure. The concentration of generated particles compared to background and coughing are shown in detail in Table 2 and in Appendix Table. Both mean and median values are reported to better describe the data collected since multiple measurements observed zero particles.

**Table 2:** Particle concentrations in laryngeal procedures and their comparison with background and coughing data

	Cold instruments	Microdebrider	CO_2_ laser	Reference	
				Background^a^	Coughing
Total particle concentration, particles/cm^3^					
Median	0.012	0.024	0.438	*0.000*	*0.036*
Mean ± SD	0.708 ± 8.047	0.030 ± 0.039	0.761 ± 2.437	*0.004* ± *0.011*	*1.601* ± *13.772*
Max	119.898	0.342	36.240	*0.144*	*195.528*
Comparisons (*p* values)					
Background	**< 0.001**	**< 0.001**	**< 0.001**		
Coughing	0.753	0.146	**< 0.001**		
< 1 μm particle concentration, particles/cm^3^					
Median	0.012	0.018	0.426	*0.000*	*0.030*
Mean ± SD	0.705 ± 8.047	0.026 ± 0.039	0.742 ± 2.436	*0.004* ± *0.010*	*1.588* ± *13.751*
Max	119.898	0.342	36.240	*0.144*	*195.510*
Comparisons (*p* values)					
Background	**< 0.001**	**< 0.001**	**< 0.001**		
Coughing	0.686	0.152	**< 0.001**		
1–5 μm particle concentration, particles/cm^3^					
Median	0.000	0.000	0.012	*0.000*	*0.006*
Mean ± SD	0.002 ± 0.004	0.003 ± 0.004	0.017 ± 0.025	*0.000* ± *0.002*	*0.012* ± *0.064*
Max	0.030	0.018	0.276	*0.018*	*1.242*
Comparisons (*p* values)					
Background	**0.004**	**0.013**	**< 0.001**		
Coughing	**0.043**	0.305	0.064		
> 5 μm particle concentration, particles/cm^3^					
Median	0.000	0.000	0.000	*0.000*	*0.000*
Mean ± SD	0.001 ± 0.002	0.001 ± 0.004	0.001 ± 0.003	*0.000* ± *0.001*	*0.001* ± *0.002*
Max	0.012	0.030	0.024	*0.006*	*0.012*
Comparisons (*p* values)					
Background	0.218	**0.005**	0.094		
Coughing	0.174	0.089	0.131		

Median, mean ± SD, and max calculated from all measured values. Measured minimum value in all laryngeal procedures and reference measurements in all size groups of particles was 0.000

*SD* standard deviation, *Max* measured maximum value

*p* values compared with aerosol concentrations of background were calculated using one-tailed unpaired-samples *t* test; for comparisons with coughing reference, two-tailed unpaired *t* test was used. *p* values < 0.05 were considered significant and presented bolded. Mean differences with 95% confidence intervals are presented in Appendix Table

ͣBackground values presented from all operation rooms where procedures took place. For each procedure type, background was tested separately according to operation room used. Reference data is presented with Italics

**Fig. 1:**
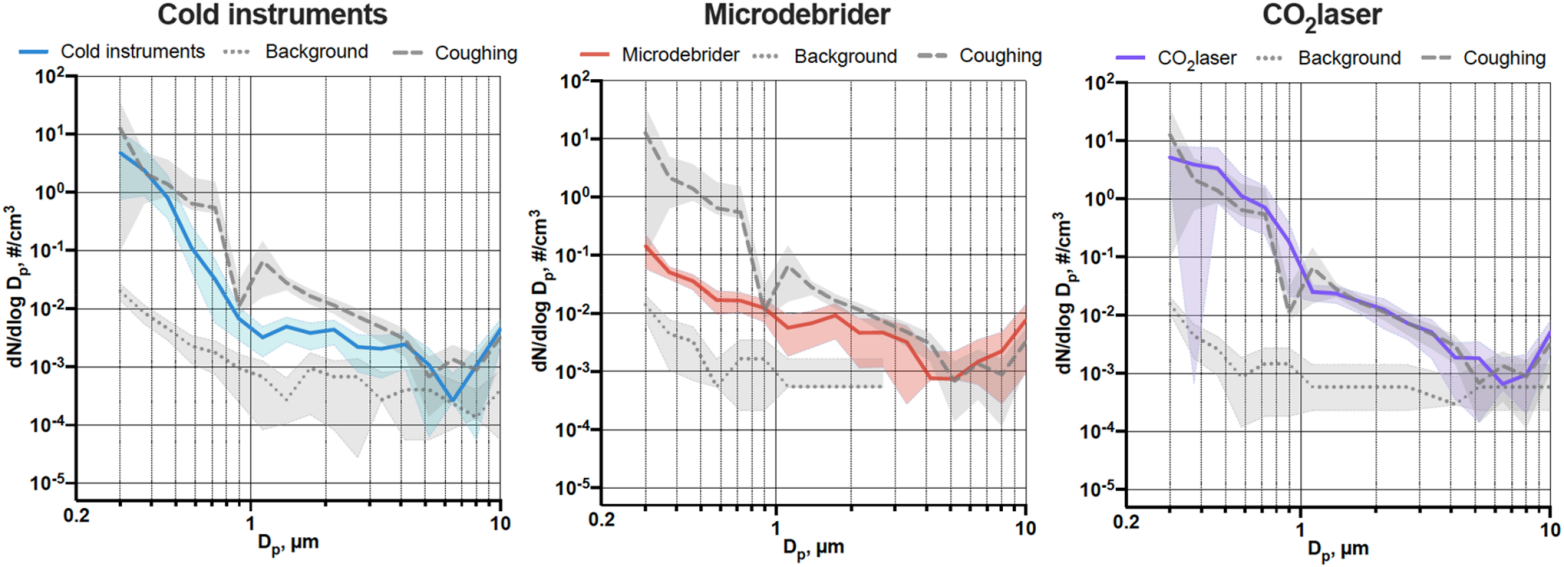
Average particle number and size distribution of measured particles when using cold instruments, microdebrider, and CO_2_ laser. Average size distribution of particles and average fractions of these aerosols in different size ranges compared with background and coughing when using cold instruments (A), microdebrider (B), and CO_2_ laser (C), expressed as mean and 95% confidence intervals. *D*_*p*_ refers to diameters of the observed particles, and d*N*/dlog*D*_*p*_ is the concentration expressed as particles per cubic centimeter

Using CO_2_ laser generated the highest concentration in both significant particle classes (< 1 μm and 1–5 μm). Average particle size distributions when using cold instruments, microdebrider, and CO_2_ laser are depicted in Fig. 1.

Significantly differences were observed between the operation techniques in total particle concentration (*F*(2) = 439.9, *p* < 0.001), < 1 μm particles (*F*(2) = 445.6, *p* < 0.001), and 1–5 μm particles (*F*(2) = 185.4, *p* < 0.001). In > 5 μm particles, no difference between the techniques emerged (*F*(2) = 1.955, *p* = 0.142). For pairwise comparisons between instruments/techniques, see Table 3.

**Table 3:** Differences in particle concentrations between laryngeal operation techniques (laser, *n* = 8; microdebrider, *n* = 4; cold instruments, *n* = 10)

		CO_2_ laser–cold instruments	Microdebrider–cold instruments	Microdebrider–CO_2_ laser
	Total particle concentration, particles/cm^3^			
	Difference (95% CI)	1.80 (1.65 to 1.95)	0.47 (0.22 to 0.72)	− 1.33 (− 1.56 to − 1.10)
	*p* value	**< 0.001**	**< 0.001**	**< 0.001**
	< 1 μm particle concentration, particles/cm^3^			
	Difference (95% CI)	2.02 (1.85 to 2.18)	0.49 (0.21‒0.77)	− 1.53 (− 1.78 to − 1.27)
	*p* value	**< 0.001**	**< 0.001**	**< 0.001**
	1–5 μm particle concentration, particles/cm^3^			
	Difference (95% CI)	1.80 (1.57 to 2.03)	0.40 (0.02 to 0.79)	− 1.39 (− 1.75 to − 1.04)
	*p* value	**< 0.001**	**0.040**	< **0.001**
	> 5 μm particle concentration, particles/cm^3^			
	Difference (95% CI)	0.15 (− 0.03 to 0.32)	0.114 (− 0.18 to 0.41)	− 0.03 (− 0.30 to 0.24)
	*p* value	0.119	0.633	0.956

Results from one-way ANOVA post hoc Tukey HSD test for multiple pairwise comparisons, adjusted by technique used for the laryngeal operation

*p* value reported after adjustment for multiple comparisons and the statistically significant results are bolded

**Fig. 2:**
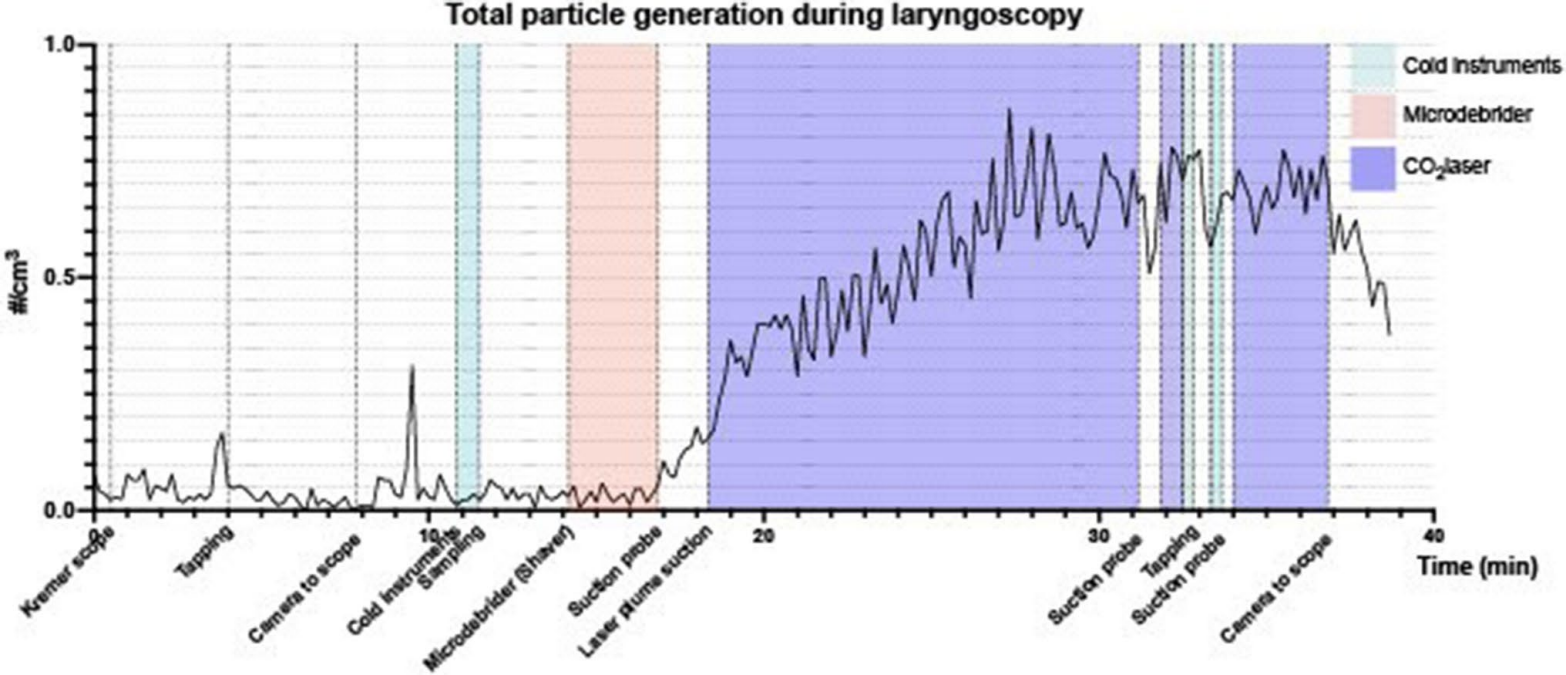
Particle generation as total particle concentration/cm^3^ during an example laryngoscopy (endoscopic excision of lesion of larynx). Particle concentration measured with 10 s scale interval. Periods when using cold instruments, shaver, and CO_2_ laser are color-coded, and starting points for the rest of the procedures are marked under the *x*-axis. Operating room information: 58.5 m^2^, 140 m^3^, temp. 20.5 °C, RH 37.5%, room air exchange 30 times/h

We also measured an operation that included all three techniques compared in this study. Figure 2 demonstrates the total particle concentration from an example laryngological procedure.

## Discussion

We showed with a large number of measurements and datapoints that the use of CO_2_ laser during microlaryngeal surgery generates a significant amount of aerosol, and thus, also increases the risk of infection for OR staff by an airborne pathogen such as SARS-CoV-2, influenza viruses, or mycobacteria [25, 26]. By contrast, use of cold instruments or microdebrider resulted in lower generation of aerosols. Based on our results, these techniques can be regarded as aerosol-generating as such, compared with background, but they should not be considered high-risk AGPs since the concentrations are low and do not exceed those observed during coughing. Our findings are valuable when planning elective surgeries or making decisions regarding the use of personal protective equipment during different laryngeal surgeries, especially during the COVID-19 pandemic or other epidemics caused by airborne pathogens. In clinical practice this means for example: (1) replacing the CO_2_ laser with cold instruments or microdebrider if possible, such as in endoscopic excision of laryngeal papillomatosis, (2) minimizing CO_2_ laser uptime when used, and (3) using FFP2 or FFP3 respirators specially in procedures where CO_2_ laser is used.

CO_2_ laser procedures are almost the only group of procedures that have been experimentally shown to produce aerosols [27]. Genangeli et al. observed aerosols from smoke samples when using CO_2_ laser to cut both soft and hard tissues in laboratory conditions [28]. Our results are in line with these earlier findings but also provide more detailed quantitative information on the number and size distribution of the aerosols produced.

We chose the size distribution measured based on airborne pathogens, such as mycobacteria, having been shown to spread especially efficiently in particles smaller than 5 μm [29, 30]. Previous studies have also confirmed that smoke, vapor, and other particles generated by CO_2_ laser can carry human papilloma virus (HPV) DNA [31, 32]. When combining these two facts and our finding that CO_2_ laser produces high concentrations of small (< 5 μm) particles, one could assume that other pathogens might also be found in aerosols generated by CO_2_ laser ablation of infected tissue. In addition, we know that time plays a significant role in pathogen exposure and infection risk. In our results, it is noteworthy that the laser procedures generating the most aerosol particles were also the longest in duration, emphasizing their role as a risk measure. However, based on our study we can only speculate the potential infectivity of transmitted pathogens; more research is warranted to address this properly.

Use of microdebrider has also previously been categorized as a potential AGP [16]. However, clinical evidence for aerosol production is weak, and it has only been evaluated during cadaveric endonasal simulation, not during real-life operations [23]. In cadaveric endonasal surgery, Workman et al. did not observe generation of 1–10 μm particles when using microdebrider. Moreover, they did not measure particles with a diameter of less than 1 μm. In our study, we observed small increases of particles when using the microdebrider compared with background, but no significant increase compared with coughing. Concentration of aerosols generated during coughing has been regarded as a benchmark for clinically significant aerosol generation during surgical procedures [9–11]. This limit was also used in this study as a limit for significant aerosol generation. When combining the findings of Workman et al. in cadaveric endonasal surgery and our results, the microdebrider should probably not be counted as an aerosol-generating instrument.

As with the microdebrider, aerosol generation when using cold instruments has previously only been studied in the context of cadaveric endonasal surgery. Workman et al. did not observe an increase in the concentration of 1–10 μm particles [23]. However, some controversial results also exist. Sharma et al. reported a significant increase in small (0.3–0.37 μm) particles during cadaveric endonasal surgery. They also reported that the production of particles with cold instrumentation was higher than when using microdebrider in the same operation [24]. Findings by Sharma et al. concerning cold instruments are in line with our results; the concentration of particles produced during cold instrumentation was not significantly higher than that produced during coughing, indicating that the use of cold instruments is not a high-risk AGP. We, did, however, observe relatively high concentrations of small (< 1 μm) particles, which may pose a relative risk of infection.

Microdebrider’s ability to not produce aerosol particles may be associated with its suction function. In many studies, suction has been observed to reduce the number of small particles in air [17, 24]. However, opposite findings also exist [16]. The controversial findings may be due to, for example, dirty or incorrectly installed filters of the suction device. Thus, proper functioning of the suction and the whole device is important.

Microdebrider application is a highly valuable option in removal of laryngeal papilloma relative to CO_2_ laser during the COVID-19 pandemic or epidemics with similar characteristics. However, it is not as valuable as CO_2_ laser in removal of minimal vocal fold edge lesions such as polyps and fibrotic lesions. In such circumstances, risk analysis is mandatory with screening for infection symptoms and possible testing 2 days before the operation [33]. Each operation should be preceded by a risk analysis to examine potential risks associated with the methods available to enable selection of the optimal approach.

This is the first study measuring aerosol generation during laryngeal procedures on real patients in the OR and comparing different laryngeal surgery techniques based on aerosol production. We measured the particle concentration and size distribution from the same distances used by OR staff, indicating that the results reliably reflect the dose of particles to which the staff are exposed. However, our setting does not indicate the total amount of produced aerosols due to the localization and use of a single OPS. The placing of the OPS also varied a little between the measurements (on average 136 cm from the head of the patient, range 110–230 cm) which can regard as a limitation of this study. However, we measured only small particles which can stay in the air for long periods of time and travel long distances which reduces the role of the device location as a whole. In addition, in our earlier study we have shown that during coughing aerosol concentrations do not change significantly as the distance of OPS changes. One of the strengths of this study is the excellent documentation of the surgical steps. Our OPS device registered all particles in the size range of 0.3–10 μm, but we did not measure the production of droplets > 10 μm. In laryngeal surgeries, the surgeon has instruments inside the patient’s respiratory tract and the patient is already relaxed and anesthetized. Accordingly, there is significantly lower risk of droplet exposure due to the absence of coughing. Nevertheless, mucus expressed in the patient’s airway above the level of the intubation tube cuff is removed by suction when needed. This differs significantly from the conditions for example during flexible laryngoscopy performed on awake patients. Therefore, our results cannot directly be applied to the overall risk assessment of voice patients examined in outpatient clinics or treated in office-based phonosurgery. There was a significant difference in the duration of the compared procedures (cold instruments 40.5 min, microdebrider 13.5, 105.2 min). This is due to the real-life nature of this study. However, even in the shortest measure, microdebrider, the number of datapoints is 81. In addition, the observed differences between procedures (CO_2_ laser vs. cold instruments and microdebrider) were large and statistically significant (*p* < 0.001) so the differences between groups do not distort the results.

Clinically significant aerosol production has not been determined accurately in the literature. In this study, we used both OR background and coughing as references for aerosol production. The procedures in which the concentration of particles produced significantly exceeded the number of particles produced during coughing were interpreted as AGPs. This classification was based on the determination of the World Health Organization and earlier studies that have used coughing as a benchmark for high aerosol production. Because of the quantitative nature of our results, they can be applied to different viruses and interpreted again as knowledge about the infectivity of airborne viruses increases.

## Conclusion

Knowledge of aerosol generation is important not only during the COVID-19 pandemic but also with regard to other airborne diseases such as tuberculosis and influenza. According to our findings, using CO_2_ laser during general anesthesia is highly aerosol-generating, while using cold instruments and especially microdebrider for tissue removal generate less aerosol.

In clinical practice, FFP2 or FFP3 respirators are recommended when using CO_2_ laser if the prevalence of COVID-19 is high, the patient has a respiratory infection, or other epidemic of airborne pathogens is recorded in the area. The risk for infection can also be reduced significantly using microdebrider or cold instruments in tissue removal instead of CO_2_ laser in clinical situations when possible.

## Data Availability

The data that support the findings of this study are available from the corresponding author upon reasonable request.

## Appendix

**Table:**

	Mean difference (95% CI)	
	Background	Coughing
Cold instruments		
Total particle concentration	1.039 (0.648 to 1.461)	− 0.072 (− 0.575 to 0.431)
< 1 µm particle concentration	1.003 (0.582 to 1.455)	− 0.097 (− 0.605 to 0.411)
1–5 µm particle concentration	0.612 (0.202 to 1.046)	− 0.485 (− 1.002 to 0.032)
> 5 µm particle concentration	0.226 (-0.305 to 0.857)	0.398 (− 0.149 to 0.944)
Microdebrider		
Total particle concentration	1.016 (0.718 to 1.315)	− 0.273 (− 1.029 to 0.483)
< 1 µm particle concentration	0.921 (0.544 to 1.298)	− 0.317 (− 1.076 to 0.441)
1–5 µm particle concentration	1.129 (0.294 to 1.965)	− 0.443 (− 1.248 to 0.361)
> 5 µm particle concentration	1.427 (0.597 to 2.256)	0.783 (− 0.029 to 1.596)
CO_2_ laser		
Total particle concentration	2.065 (1.852 to 2.279)	0.822 (0.461 to 1.182)
< 1 µm particle concentration	2.091 (1.873 to 2.309)	0.867 (0.505 to 1.229)
1–5 µm particle concentration	1.591 (1.457 to 1.724)	0.245 (− 0.102 to 0.592)
> 5 µm particle concentration	0.294 (− 0.147 to 0.735)	0.341 (− 0.088 to 0.770)

Mean difference and 95% CI calculated from log-normalized values

*CI* confidence interval
